# Newly established tumourigenic primary human colon cancer cell lines are sensitive to TRAIL-induced apoptosis *in vitro* and *in vivo*

**DOI:** 10.1038/sj.bjc.6603835

**Published:** 2007-06-05

**Authors:** E Oikonomou, K Kothonidis, G Zografos, G Nasioulas, L Andera, A Pintzas

**Affiliations:** 1 Laboratory of Signal Mediated Gene Expression, Institute of Biological Research and Biotechnology, National Hellenic Research Foundation, Vasileos Konstantinou Avenue 48, 11635, Athens, Greece; 2 3rd Department of Surgery, G. Genimatas General Hospital of Athens, Mesogion Avenue 154, 15669, Athens, Greece; 3 Molecular Biology Research Center HYGEIA “Antonis Papayiannis”, 4 Erythrou Stavrou and Kifissias Avenue, 15123, Athens, Greece; 4 Laboratory of Cell Signaling and Apoptosis, Institute of Molecular Genetics, Czech Academy of Sciences, Videnska 1083, CZ-14220 Prague 4, Czech Republic

**Keywords:** primary colon cancer cells, TRAIL, FASL, invasion, FACS, xenografts

## Abstract

Most data on the therapeutic potential of tumour necrosis factor-related apoptosis-inducing ligand (TRAIL) as well as resistance to FAS ligand (FASL) in colorectal cancer have come from *in vitro* studies using cell lines. To gain a clearer understanding about the susceptibility of patient tumours to TRAIL and FASL, we derived primary human cancer epithelial cells from colon cancer patients. Characterisation of primary cultures PAP60 and MIH55 determined their highly proliferating advantage, transforming capability and tumorigenicity *in vitro* and *in vivo*. Although FASL treatment appeared to cause little apoptosis only in the PAP60 primary culture, increased apoptosis independent of p53 was observed in both primary PAP60 and MIH55 and control cell lines Caco-2, HT29 and DLD-1 after treatment with SuperKiller TRAIL. Expression analysis of death receptors (DR) in the original parental tumours, the primary cultures before and after engraftment as well as the mouse xenografts, revealed a significant upregulation of both DR4 and DR5, which correlated to differences in sensitivity of the cells to TRAIL-induced apoptosis. Treating patient tumour xenograft/SCID mouse models with Killer TRAIL *in vivo* suppressed tumour growth. This is the first demonstration of TRAIL-induced apoptosis in characterised tumorigenic primary human cultures (*in vitro*) and antitumour activity in xenograft models (*in vivo*).

Most sporadic colorectal cancers (CRCs) are thought to develop from benign adenomas. Progression from normal epithelium through adenoma to colorectal carcinoma sequence is characterised by accumulated abnormalities to particular genes that ultimately will invade into the surrounding tissue and metastasise (Fearon and Vogelstein, 1990). *In vitro* a well-characterised model of human colon cancer cell lines exists and reflects the adenoma-carcinoma progression to facilitate research. On the other hand, there is paucity of low passage cell lines that will enable close comparison and the need for implementation with human models that will closely resemble parental primary human colon cancers is essential considering the diversity of colon cancers.

Failure in normal apoptotic pathways during carcinogenesis contributes to the resistance against anticancer drugs or radiotherapy. Prominent among cell surface molecules capable of initiating and tightly control apoptosis is the tumour necrosis factor (TNF)-related apoptosis-inducing ligand (TRAIL/Apo2L) and FAS ligand (FASL/Apo1). Tumour necrosis factor-related apoptosis-inducing ligand is a cytotoxic ligand that induces apoptosis through ligation and trimerisation of the cell surface functional death receptor (DR) TRAIL-R1 (DR4) and TRAIL-R2 (DR5), which activates the extrinsic apoptotic pathway. The RNA for TRAIL is expressed in most tissues of the human body. Although TRAIL is mostly a membrane-acting protein, small quantities of its soluble form can also be detected (Mongkolsapaya *et al*, 1999). The focus on TRAIL as a potential therapeutic agent became obvious owing to its differential sensitivity to observe between normal and cancerous cells (Ashkenazi *et al*, 1999; Walczak *et al*, 1999), while its major advantage lies with its ability to trigger tumour cell apoptosis in a variety of cancers independent of p53 status (Pitti *et al*, 1996; Galligan *et al*, 2005). Immunohistochemical studies have demonstrated that the proapoptotic TRAIL receptors DR4 and DR5 are expressed in normal colon mucosa as well as colorectal adenomas and carcinomas and their expression was increased in malignant *vs* normal cells. In comparison, there is a marked increase in sensitivity to TRAIL-induced apoptosis associated with progression from benign to malignant tumour with the assumption that the sensitivity to TRAIL is acquired early in colorectal tumorigenesis during the formation of the adenoma (Strater *et al*, 2002; Koornstra *et al*, 2003; Daniels *et al*, 2005; Hague *et al*, 2005). However, alterations in cell surface TRAIL receptor expression may not be the primary reason for this change.

FAS ligand, a cytokine of the same TNF family is a key molecule in normal immune function. Engagement of FAS by FASL triggers a cascade of subcellular events that result in programmed cell death, or apoptosis (Nagata, 1999). FAS is highly expressed in normal human colonic epithelial cell and its expression is progressively decreased during tumour progression from normal epithelium to adenocarcinoma in about 50% of the cases (Leithauser *et al*, 1993; Moller *et al*, 1994), whereas FASL has been shown to be upregulated early during colon carcinogenesis (Bennett *et al*, 2001). Many colon tumour-derived cell lines exhibit loss of sensitivity to FAS-mediated apoptosis *in vitro* (von Reyher *et al*, 1998; O'Connell *et al*, 2000). Reasons proposed for the decreased FAS sensitivity of colon tumour cell lines include downregulation of FAS receptor expression (von Reyher *et al*, 1998), overexpression of Bcl-2 (O'Connell *et al*, 2000) or FLICE-inhibitory protein (Ryu *et al*, 2001) and mutation of p53 (Tamura *et al*, 1995). Although this might suggest that during the transformation process, colon epithelial cells lose sensitivity to FAS-mediated apoptosis, it is unclear whether FASL upregulated in colon cancer leads to any increase in apoptosis of the tumour cells *in vivo*.

Circumvention of drug resistance via apoptosis induction by death ligands such as TRAIL and FASL has shown to be effective in preclinical models. In mouse models, TRAIL demonstrated remarkable efficacy against tumour xenografts of colon carcinoma (Ashkenazi *et al*, 1999; Kelley *et al*, 2001). Further, combinations of TRAIL and certain DNA-damaging drugs (Ashkenazi *et al*, 1999; Morrison *et al*, 2002) or in combination with chemotherapy has demonstrated its antitumour effect with prolonged survival without showing toxicity against normal tissue (Gliniak and Le, 1999; Walczak *et al*, 1999; Naka *et al*, 2002; Finnberg *et al*, 2005).

In contrast, almost no research has been conducted about the sensitivity of freshly isolated human colon cancer cells to TRAIL and FASL-induced apoptosis and the limited existing knowledge is demonstrated only by a handfull of publications. Normal colonocytes cultured as intact crypts has been previously reported to be completely resistant to TRAIL-induced apoptosis (Strater *et al*, 2002), whereas human colonic epithelia explants were sensitive to TRAIL-induced apoptosis with or without a chemotherapeutic agent (Finnberg *et al*, 2005). Moreover, an agonistic anti-FAS mAb enhanced apoptosis in primarily cultured tumour cells (Yao *et al*, 2004). Nevertheless, none of the above studies performed a detailed characterisation of the established colon cultures and nor explored their molecular characteristics. Molecular analysis and characterisation of newly established colon cell lines alone has demonstrated that tumorigenicity events are much more diverse in human colon cancer (Hanahan and Weinberg, 2000). Early attempts to establish human colon cancer cell lines met little success and only a few low passage cell lines that closely reflect the properties of the primary tumour cells have been established and characterised from primary intestinal epithelial cells (Gibson *et al*, 1999; Panja, 2000) and gastric cancers (Oh *et al*, 1999; Yokozaki, 2000). It was therefore necessary to combine all the above in a single study and investigate the sensitivity of primary human colon cancer cells to TRAIL and FALS *in vitro*.

Towards this end, pure epithelia cell lines were established from two individual patients (PAP60 and MIH55) with CRC and designated by the pathologists to be at Dukes C (T3N0M0) and Dukes B (T2N0M0) stage, respectively (Table 1). Of primary importance in the present study was the characterisation of the successful primary cultures using methods, which identified epithelia nature, cell phenotype and growth characteristics. Cell characterisation was concluded with *in vitro* and *in vivo* tumorigenic assays allowing experiments for primary cell sensitivity to TRAIL- and FASL-induced apoptosis. Sensitivity to apoptosis was correlated to the RNA expression levels of the DR4, DR5 and FAS in the established cell lines, an analysis that was extended to the primary tumours and their respective normal mucosa as well as their respective mouse xenografts. Tumour necrosis factor-related apoptosis-inducing ligand receptor analysis was verified by FACS in the primary cells before and after mouse engraftment. Finally, Killer TRAIL antitumour activity was demonstrated in colon cancer patient xenograft/SCID mouse models *in vivo*.

## MATERIALS AND METHODS

### Isolation of primary colon epithelial

Tumour samples and corresponding normal mucosa from two patients with CRC (Table 1) were obtained from the 3rd Department of Surgery, G Genimatas General Hospital of Athens in Greece after approval of the study by the hospital's ethical committee and with the written, informed consent from both patients.

At operation a portion of colon cancer was placed into complete D-MEM medium (containing 10% foetal bovine serum (FBS), 200 U ml^−1^ penicillin, 200 *μ*g ml^−1^ streptomycin and 5 ml non-essential amino acids) supplemented with 2.5 *μ*g ml^−1^ amphotericin B, 100 *μ*g ml^−1^ gentamycin and 200 mM HEPES (all from Gibco, BRL, Paisley, UK) and transported to the laboratory immediately on ice. The freshly resected tissue was cut into small pieces of <3 mm using cross scalpel technique (Cutfix 10 disposable sterile scalpels, Braun) and enzymatically digested with gentle agitation at 37°C for 30–60 min with 50 mg ml^−1^ collagenase type IV (Gibco, BRL), 10 mg ml^−1^ pronase E, 2000 U ml^−1^ DNAase and 0.1 mM EGTA (all from Sigma, Endorf, Germany). The dispersed tissue was mixed well by pipetting and filtered through a 100 *μ*m cell strainer (BD Falcon, San Jose, CA, USA) to remove undigested tissue fragments. Cells were washed with calcium- and magnesium-free Hank's Balanced Salt Solution (CMF-HBSS) and centrifugation at 1000 r.p.m. for 2–3 min. Colon epithelial cells were purified using discontinuous (25, 40 and 50%) Percoll (Arsham, Upsala, Sweden) gradient centrifugation (angle head rotor) for 20 min, at 600 × g. Epithelial cells were collected at the 25–40% Percoll interface using a plastic disposable Pasteur pipette (Copan, Brescia, Italy). Epithelial cells were washed twice with CMF-HBSS and centrifuged at 1200 r.p.m. for 3 min. Cell pellet was resuspended in 20% FBS complete D-MEM medium supplemented 40 *μ*g ml^−1^ gentamycin, 0.1 *μ*g ml^−1^ hydrocortisone and 2 *μ*g ml^−1^ insulin (both from Sigma) and seeded into a T-25 flask pre-coated with 10 *μ*g ml^−1^ fibronectin. Cell lines were transferred to T-75 flasks and grown to confluence before initial passage. Passage ratio was dependent on growth rate and cell lines were cryopreserved before passage 2 and up to passage 10 in complete D-MEM with 20% FBS and 10% dimethyl sulphoxide (Fluka/Sigma-Aldrich, Bushs, Netherlands) and stored in liquid nitrogen. All experiments were performed on passage 1–4 cells.

Growth rate of newly established cell lines was determined in replicate T-25 flasks seeded at 1 × 10^4^ cells 8 ml^−1^ complete D-MEM. Cells were harvested by trypsinisation and counted using a coulter counter (Coulter Corporation model Z2) after 24, 48, 72 and 144 h. Cell numbers were plotted on a log scale *vs* time and doubling times were determined during the log phase of growth.

### Immunofluorescence

For immunostaining, 5 × 10^5^ cells washed with ice-cold phosphate-buffered saline (PBS) and fixed with ice-cold methanol : acetone (4 : 1) at −20°C for 10 min. Cells were washed with PBS and nonspecific antibody binding was blocked with 5% FBS at room temperature (RT) for 30 min. Staining with pan-cytokeratin (1 : 300, Sigma, C 2562) or vimentin (1 : 600, Santa Cruz, CA, USA, sc-6260) mouse monoclonal antibodies prepared in 1.5% FBS was performed at RT for 3 h, while the secondary antibody Alexa Fluor 488 goat anti-mouse (1 : 300, Molecular Probes, Eugene, OR, USA, A 1001) prepared in 1% FBS was applied to the cells for 1 h at RT. The nuclei were stained with DNA-binding dyes Hoechst no. 33342 (Sigma, B2261) and propidium iodide (Sigma, 81845). Cells were observed under a fluorescent inverted microscope (Nikon Eclipse, T-200, Tokyo, Japan).

### Mutation analysis

Genomic DNA from the original parental primary tumours (14-PAP60 and 15-MIH55) and their respective normal mucosa (N14-PAP60 and N15-MIH55) was prepared by phenol–chloroform extraction. Each region of exon 5 (codons 126–137) and exon 8 (codons 267–289), considered hot spots for the p53 gene point mutation in both samples, was amplified by the polymerase chain reaction (PCR) method for analysis of gene mutation. The following primers were used: exon 5, sense, 5′-TTCCACACCCCCGCCCGGCA-3′, and antisense, 5′-CTGGGGACCCTGGGCAA-3′; exon 8, sense 5′-AGGACCTGATTTCCTTACTG-3′, and antisense, 5′-AAGTGAATCTGAGGCATAAC-3′. Polymerase chain reaction was performed with 250 ng of genomic DNA and PCR fragments were purified and sequenced (Biogenomica, Athens, Greece).

### Colony formation in soft agar

The anchorage-independent growth on soft agar (Deveney *et al*, 1996) was used to determine the transformation capability of these primary colon cancer cells *in vitro*. A layer (1.5 ml) of 0.5% agar in supplemented D-MEM was first set in each well of a six-well dish and allowed to equilibrate for 30 min at RT. One thousand cells/well were added to supplement D-MEM containing 0.3% agar, which was then overlaid onto the first layer of agar. Cells were incubated at 37°C for 10–14 days and medium was replaced every 2 days. Plates were stained with 0.005% crystal violet (Sigma, C 3886)/methanol for >1 h and colonies of cells that formed, larger than 0.5 or 1.2 mm, were counted under the inverted light microscope (Axiovert. 25; Zeiss, D-Jena).

### Cell invasion and migration assays

For cell invasion primary colon cells were assayed in 24-well Transwell inserts (8 *μ*m pore membranes, Coster) as described previously with modifications (Albini *et al*, 1987). Matrigel (5 mg ml^−1^) (BD) was diluted to 1.1 mg ml^−1^ in cold serum free (SF) D-MEM and 100 *μ*l of the diluted Matrigel were added into the upper chambers of the Transwell inserts (4.4 *μ*g 100 *μ*l^−1 ^well) and incubated for 4–5 h at 37°C for gelling. Gelled Matrigel was gently washed with warmed SF D-MEM and 100 *μ*l of the cell suspension in 1% FBS D-MEM was added onto the Matrigel (10^4^ cells 100 *μ*l^−1^ well^−1^). The lower chamber of the Transwell was filled with 600 *μ*l of complete D-MEM containing 5 *μ*g ml^−1^ fibronectin (Sigma), as a chemoattractant. Cell migration along with a gradient of substratum-bound fibronectin (haptotactic migration) was assayed in Transwell cell culture chambers as described previously with modifications (Wickstrom *et al*, 2004). For this assay, the lower chamber of the Transwell was filled with 600 *μ*l of D-MEM containing 1% FBS and 10 *μ*g ml^−1^ fibronectin, as a chemoattractant. One hundred microlitres of the cell suspension in 1% FBS D-MEM were seeded onto the gelatine-coated cell culture inserts (10^4^ cells 100 *μ*l^−1^ well^−1^). The 24-well Transwell was incubated at 37°C for 24–36 h. The following day, insert chambers were fixed with absolute methanol for 5 min and stained with 0.5% crystal violet/methanol for 10 min. Non-invading cells were moved with a cotton-tipped swab and only cells on the lower surface of pore filter were counted under the light microscope (× 200).

### Tumorigenicity in SCID mice

To investigate the tumorigenicity of the isolated colon cancer cell lines, cells were harvested from T-75 flasks (Sarstedt, Nümbrecht, Germany) by trypsinisation and washed extensively in PBS. One million (1 × 10^6^) cells (Caco-2, HT29, PAP60, MIH55) in a total volume of 100 *μ*l PBS were subcutaneously (s.c.) injected (21G needle) into each flank of a SCID mouse (Charles River Laboratories Inc., L'Arbresle, Cedex, France). Three female 5-week-old athymic mice were used per cell line. All animal experiments were approved by the Animal Ethics Committee of the Institute of Biological Research and Biotechnology, National Hellenic Research Foundation and procedures were according to the guidelines approved by the UKCCCR (Workman *et al*, 1998). Tumours were isolated from each condition after 2 weeks of their appearance and their weight was determined. Removed tumours had reached an average size of 2 cm.

### Cytotoxicity and apoptosis assay

For cell viability, cells were seeded into 12-well plates at a density of 1 × 10^6^ cells ml^−1^ well^−1^ and allowed to attach overnight in complete D-MEM. After overnight equilibration at 37°C, medium was replaced with fresh D-MEM supplemented with varying concentrations of human recombinant SuperKiller TRAIL (CC-TRAIL, contains two additional cysteins in the N-terminus behind the ATG codon and also contains AAs 95–281 of human TRAIL sequence) and human recombinant rhsSuperFasLigand (FASL) (200 ng ml^−1^) (ALX-522–020, Alexis). Cells were incubated for 16 h after which cells were washed with PBS and fixed with absolute methanol for 5 min. After methanol was removed, cells were stained with 0.5% crystal violet/methanol for 10 min and subsequently washed under tap water. Cells were allowed to dry overnight after which the remaining crystal violet was extracted using 30% acetic acid. Absorbance was measured at 595 nm and results presented as percentage of the corresponding control. The percentages of viable, necrotic and apoptotic cells were assessed by exposure to the DNA-binding dyes Hoechst no. 33342 (Sigma). Apoptosis was assessed by immunoblotting for PARP cleavage (1 : 1000, Santa Cruz, sc-8007) and caspase-3 activation (1 : 1000, Santa Cruz, sc-1225). Apoptotic effects were also assessed in the presence of Z-VAD-FMK (Alexis) pan-caspase inhibitor.

### Reverse transcription–PCR (RT–PCR)

A fresh specimen ⩽0.5 cm in any single direction was placed in 5 ml RNA*later* and transferred to the laboratory at 4°C. The tumour sample (about 100 mg) was homogenised in 2 ml TRIzol reagent (Invitrogen, Karlsruhe, Germany) on ice using an electric tissue grinder (ULTRA-TURRAX, type T-25; Junke and Kunkel). For cells grown in monolayer, 2 ml of Trizol reagent was added directly to the 3.5 cm Petri dish (Greiner). RNA was extracted from homogenised tissue and lysed cells according to the manufacture. The dry RNA pellet was dissolved in RNAase-free water and its concentration was estimated. The extracted total RNA (3 *μ*g – cell lines and tumour samples) was reverse transcribed into cDNA using the SuperScript™ II Reverse Transcriptase (Invitrogen) according to the manufacture. For RT–PCR amplification, 5 *μ*l of cDNA was added to a 50 *μ*l reaction containing 6 *μ*l reaction buffer (10 ×), 5 *μ*l MgCl_2_, (25 mM), 1.5 *μ*l deoxynucleotide triphosphate MIH55 (10 mM), 3 *μ*l of each corresponding 5′- and 3′- amplimer (20 *μ*M), 0.2 *μ*l *Taq* DNA polymerase (5 U *μ*l^−1^) (Invitrogen) and 26.3 *μ*l autoclaved double-distilled water. Each DNA sample was amplified using the MJ thermocycler PTC-200. Hot start conditions were used followed by initial denaturation at 95°C (3 min) and final extension at 72°C (7 min). The reaction for the DR4, DR5 and GAPDH primers (Drosopoulos *et al*, 2005) was carried through 28 cycles of 95°C (1 min, 58°C (45 s) and 72°C (45 s)). The reaction for FAS primers (Rozen and Skaletsky, 2000) was carried through 30 cycles of 95°C (1 min), 60°C (1 min) and 72°C (1 min). Intensity values were measured using Molecular Dynamics ImageQuant Software (Amersham Biosciences, Upsala, Sweden). All PCR products were normalised to GAPDH expression.

### FACS analysis

For immunostaining, 5 × 10^5^ cells were preincubated with blocking buffer (PBS containing 0.2% gelatin, 0.1% sodium azide and 20% FBS) on ice for 10 min and then incubated in the staining buffer (PBS containing 0.2% gelatin and 0.1% sodium azide) with primary monoclonal antibody DR4 (50 *μ*g ml^−1^, Alexis, HS101) and DR5 (50 *μ*g ml^−1^, Alexis, HS201) on ice for 30 min. After washing, cells were incubated in the staining buffer containing phycoerythrin-conjugated goat anti-mouse (GAM-PE) (1:500, Southern Biotechnology Associates, 1070–09). Cells were then washed, resuspended in PBS and analysed by flow cytometry. At least 25 000 viable (negative propidium iodide staining) cells were analysed for each condition.

### Mouse xenograft TRAIL study

Human colon cancer cells (DLD-1, PAP60, MIH55; 1 × 10^6^) were implanted s.c. in the flank of each mouse. Animals bearing tumours were randomly assigned to six treatment groups (five mice/group) and treatment initiated after 25 days and when representative tumours had formed. For the next 5 days, mice were given daily intravenous (i.v.) injections via the tail vein of 25 mg kg^−1^ day^−1^ Killer TRAIL (HisTRAIL, amino acids 95–281) with low amount of *Escherichia coli* endotoxins (below 5 EU/mg) purified as described by Plasilova *et al* (2002). Control groups were left untreated. After TRAIL treatment, tumour growth and mice weight were monitored every 5–6 days for 11 days. Tumours were measured using the Gage digital callipers (Kroeplin GmbH) and tumour volume was calculated with the formula V=LD × (SD)^2^/2, where V is the tumour volume (mm^3^), LD is the longest tumour diameter and SD is the shortest tumour diameter (Hylander *et al*, 2005). After day 16, animals were euthanised due to large tumour size and pieces of tumour ⩽0.5 cm in any single direction were placed in 5 ml RNA*later* subsequent RT–PCR analysis.

## RESULTS

### Isolation and establishment of human primary colon epithelial cells

**Figure 1:**
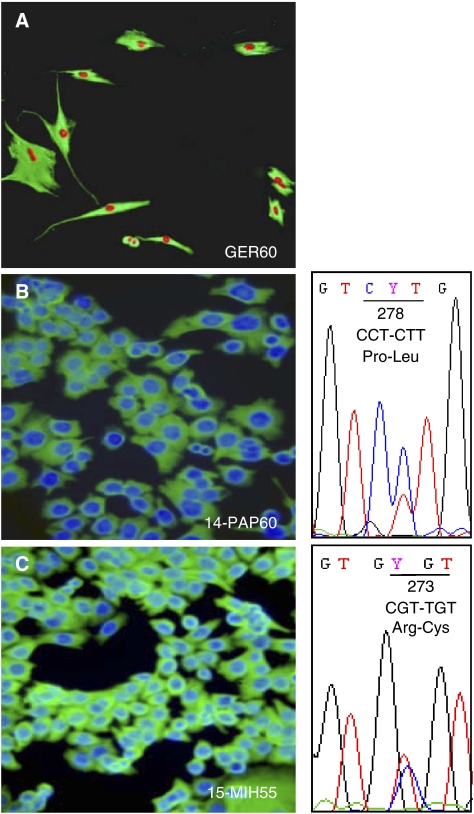
Establishment of primary human colon cancer epithelial cells by Percoll gradient centrifugation. (**A**) Immunofluorescence staining with anti-vimentin of cultures enriched with both epithelia and fibroblasts and nuclear staining with propidium iodide. (**B**, **C**) Pure epithelial cultures reacted only with pan-anti-cytokeratin and the nuclei were stained with Hoechst. The DNA sequence of the p53 gene showed for (**B**) 14-PAP60 a point mutation in exon 8 at codon 278 (transition C to T) and for (**C**) 15-MIH55 in exon 8 at codon 273 (transition C to T).

Percoll gradient-purified colon cancer epithelial cells produced cultures that contained exclusively epithelial cells only in a limited number of cases. In most occasions, cultures obtained contained polygonal-shaped cells and as determined by immunofluorescence staining with anti-vimentin, they represented fibroblast contamination within the epithelial cells. Cultures obtained from a successful Percoll gradient purification were shown to be free of fibroblast contamination by immunofluorescence staining with anti-vimentin and anti-pan-cytokeratin, where cells reacted strongly only with anti-pan-cytokeratin giving a negative reaction for anti-vimentin (Figure 1).

After plating single cells in their respective growth medium, the cells were observed to attach themselves to the tissue culture vessel with 16–24 h. At this time, the majority of cells were degenerating and/or dying, and only a few cells remained healthy. Initial primary cell cultures could be maintained for at least 4 weeks before their first passage. Early passages of both cell lines were used in all further experiments presented in this study. Primary cultures established were of epithelial origin and morphology and were characterised by patterns of cancer cell growth.

Mutation analysis for p53 indicated point mutations at codon 273 (15-MIH55) and 278 (14-PAP60) in exon 8, suggesting that mutations in the region of the DNA-binding domain (amino acids 102–292) are correlated with malignancy (Figure 1B, C).

### Cellular morphology and growth characteristics of human primary colon cells

**Figure 2:**
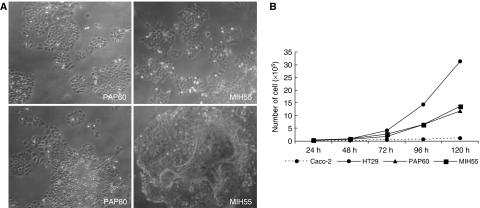
(**A**) Both primary human colon cancer cells (PAP60 and MIH55) exhibit epithelial-like morphology. After forming polarised islets during the first week of growth (initial growth), (upper panel) they showed a tendency to ‘pile-up’ to a different extend on the top of the first layer, while in advanced growth (lower panel). (**B**) Growth rate of primary and established human colon cancer cell lines. Cells were seeded at 1 × 10^4^ cell/T-25 flask and counted every 24 h for a period of 5 days.

Cellular morphology was analysed under the light microscope for potential alteration of the normal epithelial phenotype and growth characteristics. After plating and while in the first few weeks of cell proliferation, epithelial cells appeared well rounded and most single cells begun to spread creating small areas of epithelial monolayers that formed polarised islets. As cell growth advanced into the third and fourth week cells showed a tendency to ‘pile-up’ to a different extend on the top of the first layer resulting into bulks (Figure 2A).

Cell proliferation for each of the primary cell lines was determined using as controls the known growth rates of established cell lines (Caco-2 and HT29). Doubling times for PAP60 and MIH55 ranged from 15 to 18 h, respectively, resembling that of HT29 cells (18 h). Specifically, both primary cell lines PAP60 and MIH55 grew with similar kinetics as the HT29 carcinoma cells. Caco-2 intermediate adenoma cells had the slower proliferating rate and doubling time of 39 h (Figure 2B).

The anchorage-independent growth on soft agar determined the transformation capability of the primary colon cancer cells *in vitro*. Both primary cell lines PAP60 and MIH55 formed tightly packed and larger colonies as compared with control cell lines (Caco-2 and HT29) over the period of 2 weeks. Specifically, PAP60 cell line had a significantly increased ability to grow on soft agar and formed a threefold higher number of colonies as compared with MIH55 and HT29 cells, whereas the MIH55 cell line formed a similar number of colonies with the HT29 carcinoma cells (Table 2). The primary cell lines were subcultured twice before they were used in this soft agar assay. Both PAP60 and MIH55 exhibited significant transforming capabilities with PAP60 having the greatest malignant transformation.

### *In vitro* and *in vivo* tumorigenic characterisation of human primary colon cells

The tumour cell invasion ability of Matrigel in Transwell cell culture chamber assay was investigated for the two primary cell lines PAP60 and MIH55. In particular, PAP60 cells showed better invasive ability as compared with the MIH55 cells but both primary cell lines were considerably less invasive when compared with the control HT29 carcinoma cell line. As the Transwell chambers include the step of migration for tumour cells to fibronectin, the haptotactic migration ability of primary cancer cells was also investigated. In this case, MIH55 cells had an increased migratory rate compared with the PAP60 cells and resembled the migration capability of HT29 cells (Table 2).

In a further experiment, the tumorigenic potential of the two primary cell lines PAP60 and MIH55 was assessed *in vivo* by their capacity to grow tumour in mice with multiple immunodeficiencies. All mice injected with MIH55 cells developed tumours in 18 days, whereas, eight out of 12 mice developed xenografts in 25 days after injection with PAP60 cells. Both primary cell lines had similar tumour formation period that also matched tumour formation time required by HT29-positive control cells. Of note, tumour formations were the negative control Caco-2 intermediate adenoma cells for a period of 3 months (Table 3). It was also noted that PAP60 allowed for a larger tumour formation compared with the other cells, whereas the tumours formed by MIH55 were slightly increased compared with those of HT29 cells. None of the mice presented a metastasis.

### TRAIL- and FASL-induced apoptosis

**Figure 3:**
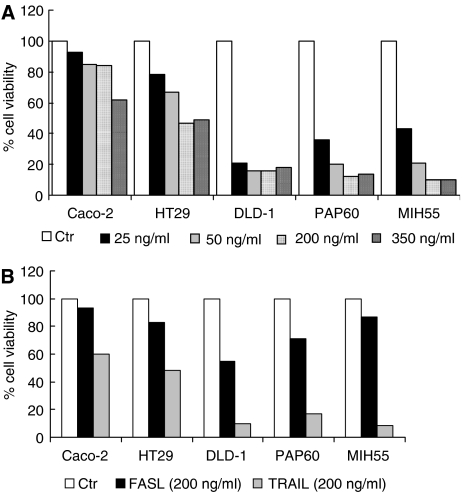
Response of human colon adenoma-carcinoma cell lines (Caco-2, HT29 and DLD-1) and primary human colon cancer cells (PAP60 and MIH55) to SuperKiller TRAIL and rhsSuperFasL treatment. (**A**) A dose response with TRAIL (25–50–200–350 ng ml^−1^) was performed to determine the optimal dose. (**B**) The cells were incubated in the presence and absence of TRAIL (200 ng ml^−1^) and (FASL 200 ng ml^−1^) for 16 h after which viability was measured. The cytotoxic effects of TRAIL and FASL treatment were measured with a standard crystal violet staining assay. Results are present as mean±STDEV of two individual experiments.

**Figure 4:**
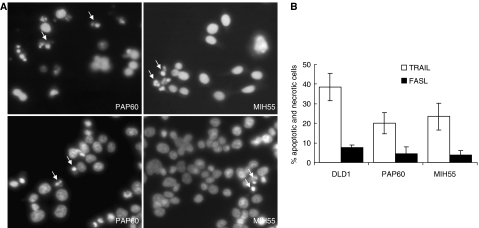
(**A**) Nuclear staining of primary colon cells PAP60 and MIH55 with Hoechst after 6-h treatment with TRAIL (200 ng ml^−1^) (upper panel) and FASL (200 ng ml^−1^) (lower panel). (**B**) Five random fields were checked for apoptotic or necrotic nuclei and indicated here by arrows.

**Figure 5:**
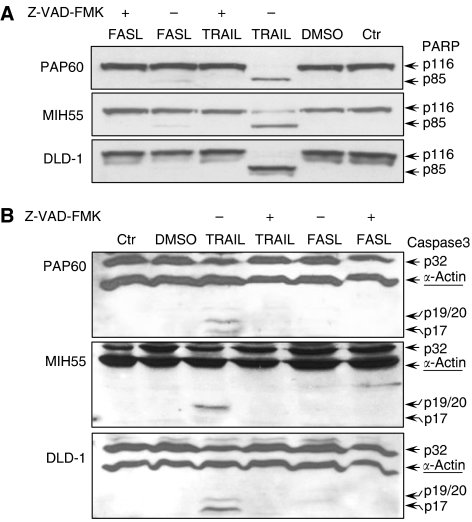
(**A**) PARP degradation and (**B**) caspase-3 activation of primary (PAP60 and MIH55) and control (DLD1) colon cells induced by TRAIL and FASL. Cells were treated for 60–120 min with TRAIL (200 ng ml^−1^) and FASL (200 ng ml^−1^) in the presence (1 h pretreatment) and absence of a pan-caspase inhibitor Z-VAD-FMK (25 *μ*M). Whole-cell lysates were checked with WB for characteristic PARP cleavage (120 min) and caspase-3 activation (60 min).

In order to examine the apoptotic effects of TRAIL and FASL in an *in vitro* system of human colorectal carcinogenesis, we subjected the freshly isolated primary colon cultures PAP60 and MIH55 and the established colon cells lines Caco-2, HT29 and DLD-1 to TRAIL and FASL treatment. Tumour necrosis factor-related apoptosis-inducing ligand (200 ng ml^−1^) and FASL (200 ng ml^−1^) doses resulted from a dose–response experiment (Figure 3A) and literature (Fiorucci *et al*, 2001), respectively. Both primary colon cultures PAP60 and MIH55 found to be highly sensitive to TRAIL (200 ng ml^−1^), as they both had significantly reduced viability within 16 h (Figure 3B) by undergoing apoptosis as detected by Hoechst staining (Figure 4), PARP cleavage and caspase-3 activation (Figure 5). The sensitivity of both primary cell lines to TRAIL resembled that of DLD-1 cells that are known to be highly sensitive to TRAIL and was similar to the sensitivity of HT29 cells that are also sensitive to TRAIL but to a lesser extent. Caco-2 intermediate adenoma cells showed no evidence of apoptosis as expected. On the contrary, only the PAP60 primary and the sensitive DLD-1 cells had reduced viability within 16 h after FASL (200 ng ml^−1^) treatment (Figure 3B) by undergoing apoptosis as detected by Hoechst staining (Figure 4).

Following FASL treatment, partial PARP cleavage was clearly detected only for the two primary cell lines, while a slight reduction of the full-length protein (p32) indicated caspase-3 activation (Figure 5). In the presence of the pan-caspase inhibitor Z-VAD-FMK (25 *μ*M), PARP cleavage and caspase-3 activation were inhibited indicating that caspase-3 activation requires protein synthesis in the sensitive TRAIL- and FASL-treated cells.

### Differential expression of DR in xenografts, respective primary tumours and derived cultures

**Figure 6:**
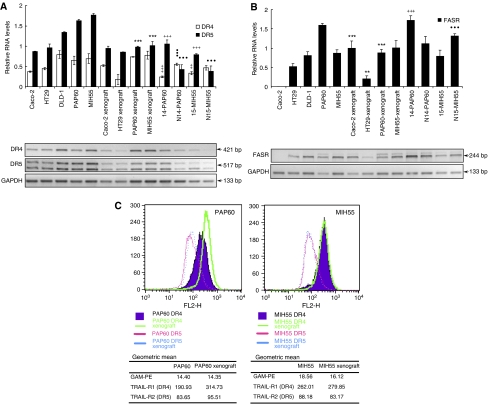
Relative RNA levels of (**A**) DR4, DR5 and (**B**) FAS in primary PAP60 and MIH55 and control Caco-2, DLD-1 and HT29 human colon cancer cell lines was performed by RT–PCR analysis. The analysis was expanded to the cell xenografts as well as the original parental primary tumours (14-PAP60, 15-MIH55) and their respective normal mucosa (N14-PAP60, N15-MIH55). The analysis was performed in triplicates and the mean±STDEV is shown. Columns, relative RNA level normalised to GAPDH. ^**^P<0.01, ^***^P<0.001 *vs* respective control cell lines in culture; ^++^*P*<0.01; ^+++^*P*<0.001 *vs* respective control cell line in culture; *P*<0.01; *P*<0.001 *vs* primary parental tumour. Student's two-tailed *t*-test was used. (**C**) Flow cytometry of DR4 and DR5 receptor expression on the cell surface in primary colon cancer cell lines PAP60 and HIM55 before and after engraftment in SCID mice. The data are presented as the log peak fluorescence intensity of untreated cells. The numbers in the first row represent the background signal when only the secondary antibody GAM-PE is used.

The expression levels of the functional TRAIL and FASL receptors were examined at the RNA level by RT–PCR. Increased levels of DR4 and DR5 RNA levels were detected in both primary cell lines PAP60 and MIH55 as compared with the Caco-2, HT29 and DLD-1 cells, whereas highest FAS expression was observed in PAP60 cells as compared with the MIH55 and control cell lines (Figure 6).

In the original parental tumours of both PAP60 and MIH55, the DR5 TRAIL receptor was upregulated, while the DR4 was downregulated as compared with the normal mucosa. When the primary tumours were transferred in culture, there was a significant increase in the DR5 receptor expression by 1.8- and 2.2-fold in both PAP60 and MIH55 cells. Same upregulation pattern was observed for the DR4 receptor, which increased its expression by 2.3-fold in both primary cultures. Interestingly, when primary cells were injected into SCID mice, RT–PCR analysis of the tumour xenografts showed significant downregulation of the DR5 that almost matched original expression levels in the parental tumours. As for the DR4, the cell culture receptor expression pattern was preserved in the tumour xenografts. For comparison purposes, same analysis was performed for the control cell lines Caco-2 and HT29, presented no significant alterations in the expression of DR4 and DR5 receptors apart from a downregulation in the DR4 receptor expression in the case of HT29 cells (Figure 6A).

FAS expression analysis showed higher expression in the PAP60 original parental tumour as compared with its normal mucosa, whereas the opposite expression pattern existed in the MIH55 parental tumour. FAS expression in the primary cultures (PAP60 and MIH55) resembled that of the parental tumours and was significantly altered in xenograft models only in the case of PAP60 primary culture where it was downregulated by twofold. In addition, FAS expression was significantly upregulated in mouse xenografts in Caco-2 intermediate adenoma cells and downregulated for in the HT29 carcinoma cells (Figure 6B).

To determine whether the actual expression levels of DR4 and DR5 receptors were altered on the cell surface before and after engraftment in SCID mice and how that compares with the RT–PCR analysis, we performed FACS analysis that confirmed the high expression levels of DR4 and DR5 before and after engraftment. Only in case of the PAP60 cells, both receptors were unregulated after engraftment in SCID mice (Figure 6C).

### Activity of killer TRAIL in a SCID mouse xenograft study

**Figure 7:**
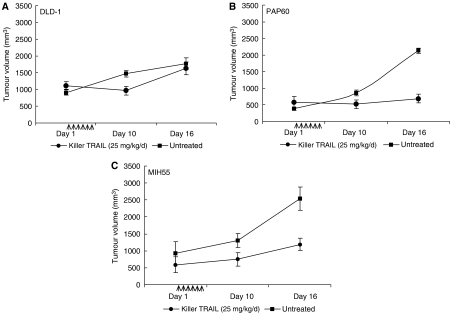
Effect of TRAIL on tumour formation. SCID mice (*n*=5/group) were injected s.c. with 10^6^ (**A**) DLD-1, (**B**) PAP60 and (**C**) MIH55 cells. Twenty-five days later, established xenografts were given Killer TRAIL (25 mg kg^−1^ d^−1^) as an i.v. bolus for 5 consecutive days or left untreated. All experiments were in compliance with the UKCCCR Guidelines for the Welfare of Animals in Experimental Neoplasia. After TRAIL treatment, tumours were measured on every fifth or sixth day for a period of 11 days. Arrows indicate the 5-day period of TRAIL treatment. Results shown are group mean±STDEV. After day 16, animals were euthanised due to large tumour size.

**Figure 8:**
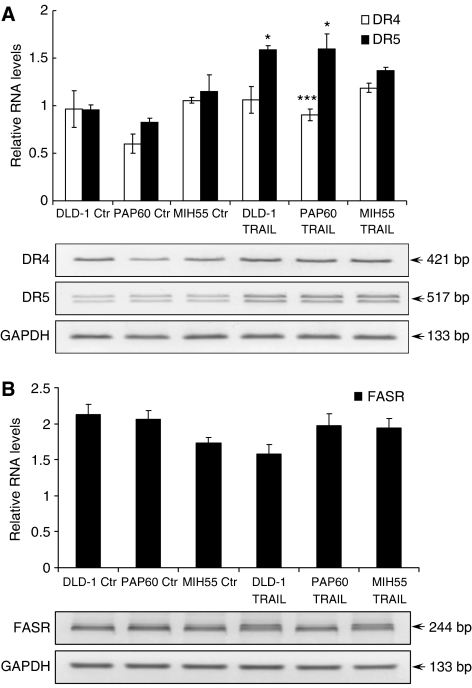
Relative RNA levels of (**A**) DR4, DR5 and (**B**) FAS in primary PAP60 and MIH55 and control DLD-1 human colon cancer cells was performed by RT–PCR analysis on established xenografts with and without TRAIL (15 mg/kg/d) treatment. The analysis was performed in quadruplicates and the mean±STDEV is shown. Columns, relative RNA level normalised to GAPDH. ^*^P<0.01, ^***^P<0.001 *vs* untreated. Student's two-tailed *t*-test was used.

In a recent study (Kelley *et al*, 2001), i.v. administration of a recombinant human Apo2L/TRAIL (30 mg kg^−1^) showed significant antitumour activity. Data from the present study demonstrate that tumour suppression was also observed to an extent following i.v. dosing of Killer TRAIL (25 mg kg^−1^). Control untreated tumours increased in tumour size and reduced their body weight over the period of 16 days, while in treatment groups tumour increase was significantly longer and body weight had increased (Figure 7 and Table 4). PAP60 and DLD-1 xenografts showed intermediate sensitivity to Killer TRAIL *in vivo* (Figure 7B) and recorded a decrease in tumour size for 5 days after which tumours continued to grow (day 16). In case of the MIH55 xenografts, there was no reduction in tumour size, only decreased rate of tumour growth was observed. No macroscopical signs of tail cytotoxicity were observed during treatment administration. RT–PCR analysis on RNA extracted from mouse xenografts with and without TRAIL treatment showed an upregulation of DR4 and DR5 only in DLD-1 and PAP60 both of which were sensitive to TRAIL *in vivo* and had a reduction in tumour size. During tumour removal, high and extensive angiogenesis was observed in both control and treatment groups (Figure 8).

## DISCUSSION

Tumour necrosis factor-related apoptosis-inducing ligand has been shown to exert enhanced apoptotic activity on tumour cells, while non-tumour cells have been reported to be resistant to TRAIL-induced death in many systems (Walczak *et al*, 1999), whereas FASL kills only sensitive FAS-bearing cells by inducing apoptosis (O'Connell *et al*, 1999; Grosch *et al*, 2001). There have been only two separate studies where TRAIL- and FASL-induced apoptosis in primary human colon cancer cells at doses that have been previously shown to be sensitive in several colon cancer cell lines *in vitro* (Yao *et al*, 2004; Finnberg *et al*, 2005). However, none of the above studies combined extensive characterisation of the primary cells. So far, the use of continuous growing tumour cell lines resulted in many data on the triggering of apoptosis by several chemotherapeutics, including TRAIL and FASL, but little is known about this process in primary human epithelia. In contrast to the continuous growing colon cell lines, human colonic cancer tissue exhibits a number of different cell types at various stages of differentiation and thus may differ in proliferation rates. Since the differences between cell lines and primary cultures are not limited to the growth rate, extensive characterisation of the primary epithelial is essential. This is the first study where characterised low passage tumorigenic primary human colon cancer cell lines PAP60 and MIH55 have been analysed *in vitro* (cell culture) and *in vivo* (SCID mice) for their sensitivity to TRAIL- and FASL-induced apoptosis administered as single agents. Both primary cell lines were highly sensitive to TRAIL-induced apoptosis (*in vivo* and *in vitro*) in contrast to the moderate sensitivity exhibited upon FASL treatment (*in vitro*) only by the PAP60 cells. Hereby, TRAIL alone induced apoptosis in the primary cultures when other studies demonstrated that combination and/or pretreatment of TRAIL with other anticancer agents was required to increase ability to induce apoptosis in both primary and established cell lines (Finnberg *et al*, 2005). Following TRAIL treatment of primary cultures, typical apoptotic morphology indicated cell death by apoptosis that was verified by PARP cleavage and caspase-3 activation. The apoptotic effect of TRAIL was independent of the p53 mutations, as both primary cell lines had mutated p53 at sites associated with the DNA-binding activity (Galligan *et al*, 2005). Tumour necrosis factor-related apoptosis-inducing ligand-induced apoptosis has been shown to be of different intensity and irrespective to the TRAIL receptor status in the different human colon cell lines. This heterogeneity in cell sensitivity is attributed to different factors such as the presence of oncogenes (Drosopoulos *et al*, 2005) or mutated caspases implicated in the downstream signalling (Kim *et al*, 2003). Similarly, FASL is also selective to the different colon cancer but to a greater extend. Cells that abundantly express FAS are nonetheless resistant to FASL-mediated apoptosis. This in turn, has been attributed to intracellular defects in FAS signalling and upregulation of the FASL when a downregulation of the FAS occurs (Houston *et al*, 2003). In the present study, only one of the two established primary cultures, MIH55, was modestly sensitive to FASL-induced apoptosis as confirmed by partial PARP cleavage and caspase-3 activation. Even though both cell lines were highly tumorigenic, PAP60 did present a better transforming capability and elevated proliferation rate but was less able to migrate through Matrigel-coated filters and form tumours in SCID mice compared with the MIH55 cell line. Regardless, PAP60 expressed higher levels of FAS that correlated to its sensitivity to FASL. According to previous reports, FAS expression was progressively decreased during tumour progression from normal epithelium to adenocarcinoma (Leithauser *et al*, 1993; Moller *et al*, 1994), which leads to conclude that other factors rather than receptor expression alone influence cell sensitisation.

Prevalent expression of DR4, 5 and FAS in both human colon cancers was confirmed by RT–PCR analysis. The analysis initiated from the original parental tumours 14 (PAP60) and 15 (MIH55) and their corresponding normal mucosa N14 (PAP60) and N15 (MIH55) and carried through the tumour-derived primary cultures PAP60 and MIH55. In both cases, DR5 was upregulated compared with the normal mucosa, whereas the DR4 was downregulated. In the primary cultures, both TRAIL receptors were upregulated, which translated to the increased sensitivity to TRAIL-induced apoptosis. When the primary cells were injected into athymic SCID mice, expression of DR4 and DR5 was significantly downregulated to levels comparable with those initially observed in the original parental tumours. Subculturing or transfer of cells away from host environment will induce them to become transformed and differentiated. This might explain differential expression of the receptors through out the different experimental models. The possibility that DR5 upregulation is a result of stress may be part of the natural homoeostasis process and the present finding could lead to better understanding of the large diversity of colon cancer tumour occurring in patients and their different response once removed from their host environment. Tumour-derived cells in culture may compromise some of their parental characteristics; therefore, it is important that experiments are performed during early passages. Even if DR5 upregulation in culture is a result of stress, expression levels in the original parental tumours cover for the sensitivity of the cells to TRAIL treatment.

On the contrary, FAS expression was upregulated in tumour 14 (PAP60) compared with the receptor downregulation observed in tumour 15 (MIH55), which also correlated to the increased sensitivity of PAP60 tumour-derived cells to FASL. Nevertheless, FAS expression pattern observed for both parental tumours was not altered in corresponding primary cultures, whereas only PAP60 downregulated FASR in mice xenografts. The p53 mutations detected in both primary cell lines might play a role, as previously suggested (Tamura *et al*, 1995), in the downregulation of the FAS observed in 15-MIH55 as compared with the normal mucosa and the decreased sensitivity of both primary cell lines (PAP60 and MIH55) to FASL treatment. Even though a number of studies tested TRAIL *in vivo* for its ability to induce apoptosis in tumour xenografts, none ever reported on the DR status or alterations in the cell lines before and while in SCID mice (Gliniak and Le, 1999; Walczak *et al*, 1999; Kelley *et al*, 2001; Naka *et al*, 2002; Finnberg *et al*, 2005; Hylander *et al*, 2005). FACS analysis confirmed the high expression levels of DR4 and DR5 in the primary cultures before and after engraftment in SCID mice. Further evidence is also provided about the functional significance of KILLER/DR5 by demonstrating that DR5 is the most differentially expressed DR between the different conditions. KILLER/DR5-mediated tumour suppression and contribution to drug-induced apoptosis importantly identify KILLER/DR5 as a promising therapeutic target for the management of CRCs (Wang and El-Deiry, 2004).

Taken together, the present study demonstrates the antitumour activity of TRAIL in a cytotoxicity (SuperKiller TRAIL) assay and xenograft models (Killer TRAIL) of primary CRC after i.v. administration, which was correlated to DR upregulation and provides estimates of the disposition of TRAIL in humans. Other apoptosis-inducing members of the TNF family carried great promise as anticancer agents at the cost of severe toxicities toward normal tissues have hampered their use as cancer therapeutics. Administration of agonistic anti-FAS antibodies or recombinant human FASL to rodents results in lethal liver damage (Ogasawara *et al*, 1993; Tanaka *et al*, 1997). The absence of macroscopic toxicity using low TRAIL concentrations (25 mg kg^−1^) is encouraging for further bioactivity and pharmacokinetic studies to implement present findings. Combination of TRAIL with chemotherapy managed to reduce cytotoxicity (Naka *et al*, 2002; Finnberg *et al*, 2005). Therefore, introducing in the *in vivo* studies promising synergistic to TRAIL agents such as Quercetin that have been proved efficient *in vitro* in our lab (Psahoulia *et al*, unpublished data) may lower cytotoxicity by reducing even more TRAIL concentrations leading to more efficient targeted therapies.

In the early part of these studies, it proved difficult to obtain a cell isolation with good viability that was enriched with pure epithelial cell. The success in culturing human colon epithelial cells is likely due to several factors like the clinical staging of the tumour. Primary cancers that had demonstrated metastasis either to regional nodes (Dukes Stage C) or distant sites (Dukes Stage D) or originated from the right side of the colon present better chances of survival and successful culture (McBain *et al*, 1984). Regardless protocol adaptations, the success rate remained low where only two out of five (40%) colon cancer biopsies survived to be of pure epithelial origin. The procedure involved tissue digestion for not more that 30 min with an enzyme cocktail that contained collagenase as a primary enzyme, pronase E to intensify the action of collagenase, as alone in high concentration was fatal for the cells and DNAse. Collagenase alone and longer incubation periods proved unsuccessful. The presence of EGTA helps to reduce rapidly the local (Ca2+)i providing cells with damaged cell membranes to better attachment capability. Percoll gradient purification was used for epithelial isolation and was far more successful than using culture medium were L-Valine was substituted for D-Valine in the presence of which only cancer cells can continue grow. Isolated cancer epithelial was seeded on fibronectin-coated vessels as the isolation procedure heavily damages the cell membrane reducing the attachment ability of the cells down to 6%. Successful long-term cultures of primary cancer colonic epithelial will contribute to better understanding of the function of human disease and CRC in specific. The established primary cell lines provided an essential experimental model for testing TRAIL- and FASL-induced apoptosis and receptor expression reflecting the great diversity that exits in colon cancer.

### Future therapeutics

For colon carcinomas, the combination of subthreshold doses of recombinant TRAIL with existing chemotherapeutic agents resulted in a substantial positive interaction, completely eliminating the tumours in some animals. These results look encouraging, although in the absence of human clinical testing, there is no certainty that the positive results achieved in animal models will translate to the complexities of the true human disease. Towards this end, primary colon cultures of derived cells presented in this study or explants investigated elsewhere may provide findings that resemble the human nature.

## Figures and Tables

**Table 1:** Patient information

	**14–PAP60**	**15–MIH55**
TNM	T3N0M0	T2N0M0
Location	Sigmoid	Rectum
Familial history	—	—
Histology	Middle differentiation	Middle differentiation
Chemotherapy	—	—
Radiotherapy	—	—
CEA (pre-op)	16.9	1.9
C 19-9 (pre-op)	5.4	4.6

Patient background and diagnosis as determined by the histopathologist.

**Table 2:** *In vitro* properties

	**Caco-2**	**HT29**	**PAP60**	**MIH55**
Colony formation (CFU)	5.95±3.46	138.9±30.3	279.8±39.5	210.1±39
Matrigel transwell invasion	8.3±2.24	168.3±20.7	84.2±19.4	64±20.7
Transwell fibronectin migration	18±1.78	172.3±16	145.7±17	169±11.7

The ability of primary control colon cancer cells to form colonies in 0.3% soft agar and the invasive and migratory ability through Matrigel-coated membranes and transwell filter membranes, respectively. The values are mean±STDEV of two individual experiments performed in duplicates.

**Table 3:** *In vivo* properties

	**Caco-2**	**HT29**	**PAP60**	**MIH55**
Tumour formation in days	92.5±16.2	21.5±9.1	25±4.2	18±0.01
Tumour number	6/9	9/10	8/12	6/6
Tumour mass (g)	0.19±0.1	0.37±0.007	0.69±0.3	0.42±0.01

Tumour formation efficiency of primary and control colon cancer cells in SCID mice after 3 months. Approximately, 10^6^ cells were subcutaneously injected into two flanks of three SCID mice and monitored for tumour formation over time. The values are means±STDEV of two individual experiments performed in triplicates.

**Table 4:** Group average tumour weight

	**DLD-1**		**PAP60**		**MIH55**	
	**Untreated**	**TRAIL 200 mg kg** ^**−1**^ ** day** ^**−1**^	**Untreated**	**TRAIL 200 mg kg ** ^**−1**^ **day** ^**−1**^	**Untreated**	**TRAIL 200 mg kg ** ^**−1**^ **day** ^**−1**^
Day 0	16.75±1.7	12.75±1.7	16.20±1.1	15.40±2.0	18.25±2.1	15.75±1.5
Day 8	14.25±2.1	13.25±1.2	15.00±1.2	15.10±1.9	16.75±3.0	16.10±0.9
Day 11	14.25±2.1	13.75±2.1	14.00±1.2	15.50±1.8	15.50±2.1	16.75±1.3
Day 14	14.75±1.3	12.75±1.8	13.80±0.8	15.40±1.9	14.75±2.1	15.75±1.8

Data are group average tumour volumes±STDEV (*n*=5 mice/group).
