# Newly established tumourigenic primary human colon cancer cell lines are sensitive to TRAIL induced apoptosis *in vitro* and *in vivo*

**DOI:** 10.1038/sj.bjc.6604137

**Published:** 2007-12-11

**Authors:** E Oikonomou, K Kothonidis, E Taoufik, E Probert, G Zografos, G Nasioulas, L Andera, A Pintzas

**Correction to**: British Journal of Cancer (2007) **97**, 73–84. doi: 10.1038/sj.bjc.6603835

The authors of the above paper would like to add two author names to the list originally published in Volume 97, Number 1; the full author list is given above, including E Taoufik and E Probert.

